# Standardized intermittent shoreline boat electrofishing for slow-flowing rivers in Sweden

**DOI:** 10.1016/j.mex.2024.102907

**Published:** 2024-08-13

**Authors:** Joacim Näslund, Thomas A.B. Staveley, Erik Petersson

**Affiliations:** Department of Aquatic Resources (SLU Aqua), Swedish University of Agricultural Sciences, Stångholmsvägen 2, SE-178 93 Drottningholm, Sweden

**Keywords:** Electric fishing, Fish surveys, Electrofishing boat, Environmental monitoring, Protocol, Swedish standardised intermittent shoreline boat electrofishing, version 1 (S-SISBE v.1)

## Abstract

Electrofishing is a common method for sampling fish in rivers. In Sweden, electrofishing has a long history, dating back to the 1950s, but the vast majority of surveys have been conducted by wading in shallow river stretches, leaving a data gap for non-wadable rivers. Boat electrofishing allows for surveys in deeper river sections, but limited numbers of operational electrofishing boats have led to standardisation of methods not being prioritized within Swedish water management. This method protocol describes the first Swedish standardised method for boat electrofishing, based on intermittent shoreline sampling in larger slow-flowing rivers. The paper describes:

• General methodology for boat electrofishing operation
• Data collection protocols
• Discussion of current caveats for the method

In the future, the methodology will be amended to cover a wider array of river types (e.g. faster flowing river sections). Hence, readers are advised to look for updates to the protocol.

## Graphical abstract

**Figure:**
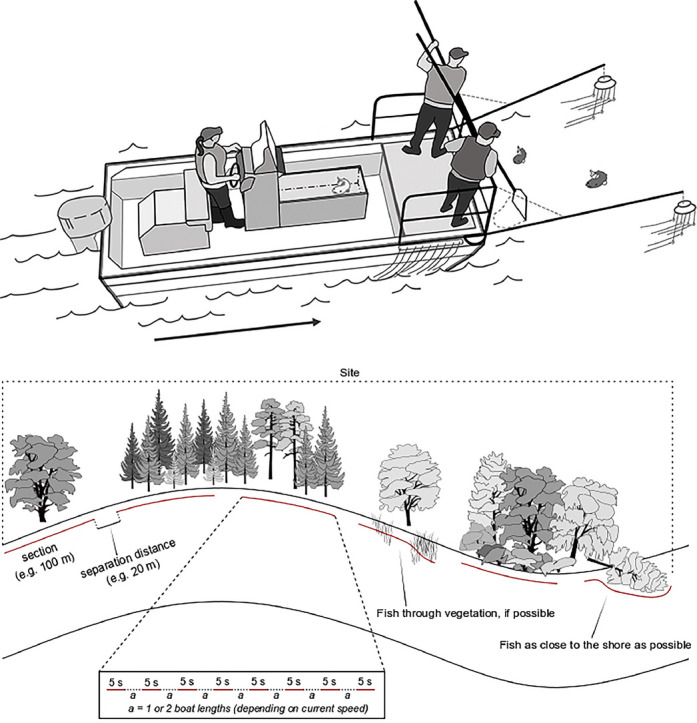

Specifications table

**Table:** 

Subject area:	Agricultural and Biological Sciences
More specific subject area:	Fisheries Science
Name of your method:	Swedish standardised intermittent shoreline boat electrofishing, version 1 (S-SISBE v.1)
Name and reference of original method:	*NA*
Resource availability:	*NA*

## Method details

### Background

This paper constitutes an English language summary of the first Swedish standardised method for boat electrofishing, developed at the Department of Aquatic Resources, Swedish University of Agricultural Sciences. This project was financed by the Swedish Agency for Marine and Water Management (‘SwAM’) and has resulted in a detailed report, in Swedish, of the method description [1] as well as a Swedish summary of results from test fishing in a report by Näslund et al. [2].

While wading electrofishing has been conducted in Sweden for over 70 years [3], boat electrofishing surveys have only been conducted occasionally since the early 2000s [2,4]. At present, only a handful of boat electrofishing operators exist in Sweden [2]. Until 2022, no standardised methods existed.

The Swedish standardised method described here aims to produce comparable boat electrofishing surveys, specifically directed towards environmental monitoring of the fish fauna in Swedish slow-flowing rivers. The main purpose for this environmental monitoring is classification of ecological status in riverine water bodies (defined segments of rivers) following the Water Framework Directive (2000/60/EG) of the European Union [5]. Slow-flowing rivers suitable for boat electrofishing range largely in e.g. size, water turbidity and colour, and channel morphology, which means that the method needs to be applicable over a wide range of river types. Given that deeper areas in rivers are relatively poorly surveyed by boat electrofishing and the effective fishing depth is limited to maximally a few meters, further reduced by turbid conditions in lowland rivers, the method focuses on fishing along the shoreline of the rivers. This limits the sampled riverine habitats to near-shore habitats, but it is assumed that the thereby sampled fish fauna can represent the ecological conditions at a given site, even though the whole fish community of the river section is not represented in the catches. While some methods stratify strips/sections over the whole river area [6], other methods rely on shoreline operation for similar reasons as outlined above [7,8]. Shoreline operation has been evaluated to give sufficient information for ecological status assessment based on the catch [9] and is the recommended method for evaluation according to the pan-European Fish Indices (EFI/EFI+) [10,11]. However, it is important to stress that proper assessments of ecological status requires a sampled distance that covers a representative range of near-shore habitats for the river. As far as conceivably possible, the method was developed in line with the European (CEN) standard for fishing with electricity (EN 14011:2003). However, this CEN standard is mainly focused on wading electrofishing, with boat-based methods only being covered in brief. Hence, some adjustments had to be made which deviate from the specified standard, since the specifications do not fit with the boat electrofishing method or the situations where it is applied.

### General methodology

**Fig. 1:**
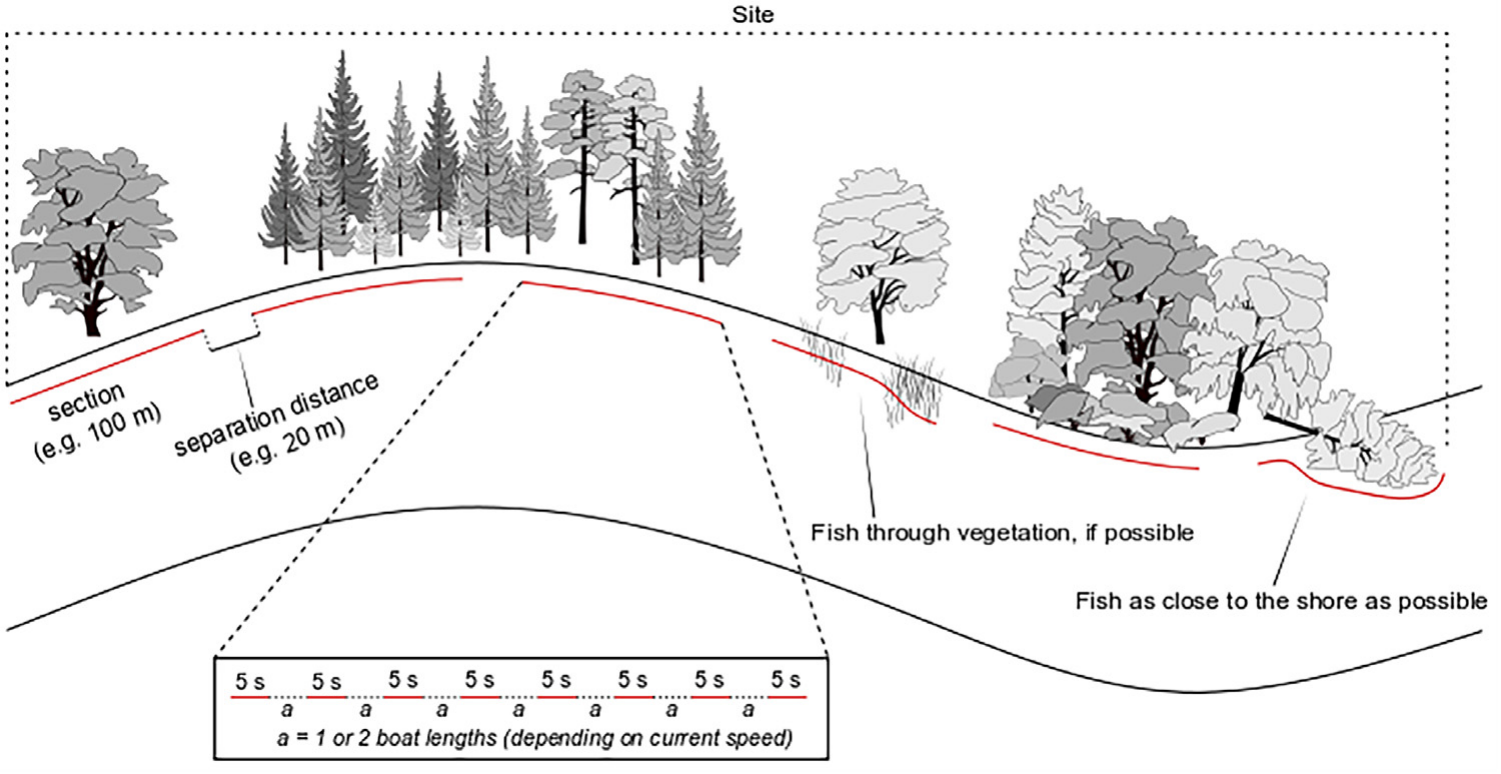
Generalised illustration of the operation over a site. The site is divided into sections, separated by separation distances. Each section is fished intermittently, using 5-s shocks between which the boat is moved 1 or 2 boat lengths (*a)*, depending on the current velocity (1 boat length under slower flow conditions, 2 boat lengths under faster flow conditions).

The general methodology rely on shoreline strip sampling (or parallel intermittent operation, following terminology specified by Grant et al. [12]). The fished target area is called a ‘site’ and is the unit at which analyses and evaluation is applied. Within a site, a general transect is outlined through the site, along one or both shorelines of the river. The site transect is divided into a number of sections (length of sections is optional, but fixed lengths are recommended), which are separated by unfished sections (‘separation distances’, Fig. 1; optional but recommended) to extend the fished stretch of the river. Each section is fished in a systematic way, where electricity is turned on for 5 s at a time while driving the boat slowly forward, in the same direction as the current (i.e. downstream). Each 5-second electric shock is separated by moving the boat one or two boat lengths, during which electricity is turned off:

• **Shock duration:** The 5-second shocks are motivated by an aim to reduce the risk of injuring the fish, which increases with longer exposure to electricity due to increased risk of coming too close to an anode.
• **Distance between shocks:** A movement of one whole boat length between shocks is suitable for slow flowing conditions and two whole boat lengths is used when the current velocity is higher, to allow for some brief pause between shocks where netting personnel can empty the nets and the driver of the boat can make notes in protocols. Usage of boat length as the reference for separation distances, instead of e.g. universal distance measures or time intervals [12], is used to make it easier for the boat driver whilst operating the boat.

The intermittent operation is motivated by observations that continuous power output will herd the fish in front of the boat, at the edge of the electrical field but outside of the attraction zone and out of reach for the netting personnel [2,12]. The boat is driven as close to the shore as possible, driving through vegetation where possible and around impassable vegetation and obstacles (e.g. overhanging or fallen trees, stones, jetties etc.) while maintaining the systematic fishing sequence (Fig. 1). This way of operating the boat through a section will lead to some deeper habitats being fished in shallow waters with obstacles (i.e. when fishing around such obstacles), but in rivers without obstacles along the shoreline (e.g. channelized stretches), the open water fish community (typically bleak *Alburnus alburnus*) is often found closer to the shore and will thereby be sampled to some extent. Open water species are, however, also caught during strict near-shore sampling [2]. While fishing during night is shown to be more efficient for at least some fish species [13], daytime fishing (i.e. in daylight; fishing during dusk or dawn is not recommended) is conducted in part to conform to safety regulations, but mainly due to the fact that the European standard requires fishing during daylight. To include fish from the young-of-the-year cohort, fishing should be conducted late summer or autumn (late July to early October).

Effective fished width of the boat needs to be recorded. If anodes are mounted on adjustable booms, the recommendation is to place them 2 m apart for optimal efficiency, based on evaluations by Miranda and Kratchovíl [14]. Settings of the electrofishing unit are adjusted (in the fished water body, pre-operation) so that the attraction distance from the anodes is 1 meter. The anode configuration then leads to an effective fished width of 4 m. If anode configuration is fixed, the effective fished width should be assessed on-site.

### Sampled distance

The protocol is adapted to be suitable to fish substantial stretches within typical Swedish river water bodies, which are constituted by relatively homogenous river sections varying in length between approximately a half kilometre to tens of kilometres. Water bodies constitute the units for water management, e.g. status classification and management action plans, following the European Water Framework Directive [5,15]. Since water bodies differ substantially in length [16], the method requires some flexibility in the required sampled distance. The sampled area should cover a representative variety of shoreline habitats for the sampled water body. Alternatively, when a waterbody contains large homogenous but discrete stretches with different habitats, separate sites could be located in each of these discrete water body stretches. In principle, a site should be as long as possible to capture the variation in habitats along the water body. The recommendation for this protocol is that each sampled site should consist of at least 1 km fished distance (counting total distances of sections, i.e. including both fished and unfished stretches of the sections, but not the separation distances between sections; Fig. 1) [2,17]. If possible, more than one site should be fished per water body. Shorter stretches could constitute a site in cases where either fish densities are high and assumed to represent the available habitats, or where the features of the water body restricts fishing (e.g. obstacles prohibiting the boat to travel further, saltwater intrusion in coastal rivers, etc.). When obstacles that may restrict navigation along the river under certain circumstances are noted within a focal area (e.g. low bridges which may only be passed during low water conditions), the focal area should be separated into two sites, one downstream of the potential obstacle and one upstream. This is to allow for at least partial replication of the fished area across years.

### Netting

The electrofishing boat should be staffed with two netting personnel, at each side of the bow of the boat. Usage of glasses with polarized lenses is recommended. Handles of the dip nets should be at least 2 m in length, have a rigid hoop large enough to catch the prevalent species (recommendation: at least 40 cm in diameter) and have a net basket deep enough to handle larger fish (recommendation: at least 30 cm) with a mesh diameter of 6 - 7 mm (or slightly larger in faster flowing waters; which should be noted in the protocol). Light-weight handles are recommended for sustained manoeuvrability over a working day. Fish are netted upon sight using calm sweeps with the nets, without prejudice towards species or size of fish, but with priority to fish close to the anode to avoid injuries on the fish. The method does not assume catching all shocked fish, and neither assumes equal catchability for different species or size classes [18,19]. Rather, it assumes that catches will be comparably biased for a given type of fished habitat. This, along with the fact that the mid-section of the river is not sampled, prohibits assessment of total biomass or densities of species. The sampling allows for comparing catches within sites to detect temporal changes in the fish community, and comparing the fish communities among sites after controlling for relevant environmental factors such as turbidity and physical habitat characteristics.

### Permits, required education, and equipment maintenance

For fishing in Swedish waters, the method requires i) an exemption from the fisheries regulation for electric fishing from the County Administrative Board responsible for the fished water body, ii) ethical permit for animal research, and iii) permit from the fishing rights owners in the fished area. In addition, all personnel handling fish should have relevant education in ethics for animal research (including fish handling and anaesthesia) and education in electrofishing. Technical competence allowing for inspecting the equipment (e.g. the boat, trailer, and electrical equipment) is required for at least one person of the operation crew. Equipment quality and -maintenance should follow national regulations on electrical equipment (Sweden: ELSÄK-FS 2016:1, which also implements the EU low voltage directive 2014/35/EU), and international safety standards (IEC 60335-2-86).

### Post-fishing disinfection of equipment

After each fishing, all equipment that has been in contact with water (including the boat engine) needs to be disinfected (e.g. by ethanol solution or other disinfectant; or complete drying, if possible) to avoid spread of waterborne diseases between water bodies. In addition, the boat should be properly cleaned from organic materials to avoid spreading invasive species.

## Recorded data

### Fish data

During surveys, all captured fish are anaesthetised, determined to species, counted and measured (maximal total body length; caudal fins folded). Data records are made collectively for all fished sections within a site. Individuals with clearly notable body deformities (head/jaw deformities and spine deformities), electrofishing related injuries and mortalities are specifically noted in association with the respective body size data. In addition, for each section it is noted whether fish where observed or not and whether fish were captured or not, to assess proportion of sections without any fish and proportion of sections where fish were detected but not captured. If uncaptured fish can be determined to species (or other taxonomic level, e.g. family), this is also noted in the protocol to provide qualitative data on species presence in the water body. Higher level of detail (e.g. fish data per section) is possible to record, but more time consuming due to the need to make breaks in the operation to process captured fish.

For each section within a given site, records are made of 1) the depth at the end of the section, and 2) effective duration of fishing (time with power output in seconds). The recommendation is that every fished section has the same length (e.g. 100 m); if not, the length of the section is also noted in the protocol. It is also possible to record number of shock operations per section, to facilitate calculating effective fished area (if not recorded, effective fished area can be estimated following steps under the heading ‘*Catch per unit effort*’ below).

### Metadata

Metadata collected during or after the survey are presented in Table 1. Several metadata variables are categorised to allow for a rapid assessment, in accordance with protocols used for standardised wading electrofishing in Sweden [20]. In addition to metadata collection, photographic documentation of a site is recommended.

**Table 1:** Metadata collected during or after surveys. Protocols: A – survey metadata; B – weather metadata and notes; C – site metadata I (geographic); D – site metadata II (environmental); E – equipment and operation metadata.

Protocol	Name	Recorded data	Type of data
A	Purpose	Explanation of survey purpose	Text
A	Client	Who ordered the survey?	Text
A	Contractor	Who performed the survey?	Text
A	Contractor contact details	Phone number and e-mail	Text
B	Cloud cover	Fraction of sky obscured by clouds in oktas; 3 classes: 1) 0–2 oktas, 2) 3–5 oktas, 3) 6–8 oktas	Ordinal
B	Precipitation	Millimetres of precipitation during the day of fishing^1^	Continuous
B	Wind	Wind affects fishing negatively or not	Binary
B	Air temperature	Air temperature in degrees Celsius during the fishing	Continuous
C	Site name	Standardised name of the survey site as defined by start and stop coordinates	Text
C	River name	Name of the river	Text
C	Water body ID	Swedish water body object identifier (‘MSCD’; format: ‘WA12345678’)	Text
C	Main catchment	Name of the main catchment, determined by the name of the downstream-most river at the sea, following the Swedish Meteorological and Hydrological Institute^3^	Text
C	Administrative region	County and municipality	Text
C	Start coordinates	Coordinates for the start position of the survey site	SWEREF99TM
C	Stop coordinates	Coordinates for the end position of the survey site	SWEREF99TM
D	Water level	Assessed water level as compared to normal flow; 3 classes: 1) Low, 2) Normal, 3) High	Ordinal
D	Water velocity	Water velocity ca 1 m below the surface, or in the middle of the water column or at 1 m depth if deeper than 2 m; measured by current velocity meter.	Continuous
D	Water temperature	Water temperature in degrees Celsius, measured ca 20 cm below the surface	Continuous
D	Water conductivity	Water conductivity in mS/m; measured by conductivity meter at the end of the	Continuous
D	Channel width	Average width of the fished river section under normal flow.	Continuous
D	Fished side of the river	Fished side of the river, facing downstream; 5 categories: 1) Left, 2) Right, 3) Both sides, 4) Mid channel, 5) Island – ‘Island’ means fishing conducted around an island within the river channel.	Nominal
D	Bottom substrate	Dominating bottom substrate, 4 categories + text option for “other”; 1) Organic, 2) Fine (clay, silt), 3) Sand, 4) Gravel/Rocks, 5) Other (fill in).	Nominal
D	Bottom topography	Overall bottom topography over the survey site; Even or Uneven (assessed by echo sounder).	Binary
D	Bottom hardness	Overall bottom substrate hardness over the survey site, 3 categories: 1) Soft (organic, fine sediment, sand), 2) Hard (gravel or rocks), 3) Mixed (substantial variation in substrate hardness)	Nominal
D	Water vegetation	Dominating type of water vegetation, 4 categories: 1) emergent plants; 2) submergent plants, 3) floating-leaf plants, 4) benthic plants.	Nominal
D	Vegetation cover	Areal cover of plants (all types of aquatic vegetation in fished section; not whole channel), 5 categories: 1) 0–5%, 2) 6–10%, 3) 11–25%, 4) 26–50%, 5) > 50%	Ordinal
D	Turbidity	Water turbidity, 3 categories: 1) Clear, 2) Turbid, 3) Very turbid	Ordinal
D	Water colour	Water colour, 3 categories: 1) clear, 2) coloured, 3) very coloured	Ordinal
D	Terrestrial environment	Dominating environment within 200 m from the river channel at the fished site, categorised as: D1 – most dominating, D2 – second most dominating, and D3 – third most dominating (types: Deciduous forest, Evergreen forest, Mixed forest, Clear-cut forest area, agricultural field, meadow/grass, heath/moor, bog, wetland, bedrock, bare fell, urban/industrial	Nominal/Ordinal
D	Riparian trees	Trees along the fished shoreline, coverage in percent of shoreline distance, 5 categories: 1) 0–5%, 2) 6–10%, 3) 11–25%, 4) 26–50%, 5) > 50%	Ordinal
D	Submerged large dead wood	Number of visible pieces of dead wood observed (length > 1 m, diameter > 0.1 m; submerged or semi-submerged; counted by netting personnel)	Count
E	Boat model	Model of the electrofishing boat	Text
E	Boat length	Length of the boat hull. Unit: meters (m).	Continuous
E	Pulsator	Model of the electrofishing pulsator unit	Text
E	Number of anodes	Number of anodes used	Count
E	Type of anode	Type of anode, 4 categories: 1) Umbrella-type, 2) Spheric, 3) Horizontal beam, 4) Other (fill in)	Nominal
E	Current type	Type of electric current, 2 categories: 1) Alternating (AC; not recommended), 2) Direct current (DC)	Nominal
E	DC frequency	If current type = “DC”, then note DC type/frequency. Categorical choice, 2 categories: 1) Straight DC, 2) Pulsed DC. If pulsed, note the used frequency. Unit: hertz (Hz).	Nominal/Continuous
E	Voltage output	Type value and maximal value (if possible, also minimum value). Unit: volt (V).	Continuous
E	Percent power	Type value and maximal value (if possible, also minimum value). Unit: percent (%).	Continuous
E	Current	Type value and maximal value (if possible, also minimum value). Unit: ampere (A).	Continuous
E	Power	Type value and maximal value (if possible, also minimum value). Unit: watt (W).	Continuous
E	Net mesh size^4^	Distance between two knots on the diagonal when the mesh is stretched.	Continuous
E	Fished direction	Fished direction in relation to flow direction, 3 categories: 1) Concurrent, 2) Counter-current, 3) no current.	Nominal
E	Light conditions	Light conditions during the operation, 4 categories: 1) Daylight, 2) Dawn, 3) Dusk, 4) Night	Nominal
E	Time	Start and stop time of operation	Time-of-day (HH:MM; 24-hour clock)

^1^ Based on data from Swedish Meteorological and Hydrological Institute: https://www.smhi.se/.

^2^ For Swedish monitoring data, Site-ID is provided by data host (Swedish University of Agricultural Sciences).

^3^ Swedish Meteorological and Hydrological Institute: https://www.smhi.se/kunskapsbanken/hydrologi/avrinningsomraden/sveriges-huvudavrinningsomraden-1.26616.

^4^ Added after the test fishing was conducted, not present in the protocol published in [1].

### Catch per unit effort (CPUE)

Catches are assessed based on CPUE, where the unit of effort corresponds to the effective operational fishing time in seconds (i.e. time with electricity output). Some problems with time-based CPUE have been identified, e.g. relating to gear saturation [19]. Given that all instructions are strictly followed and that all individual sections fished within a site have the same length, it is possible to calculate an estimate for the effective fished area, through the following steps:

1. For each fished section (see Fig. 1) of a site, divide the total operational fishing duration in seconds with the operational duration per shock (i.e. 5 s, following the method instructions; but other durations can be applied). Round down to the nearest integer (to cope with the possibility that some shocks might have been slightly longer than the specified operational shock duration). This provides the number of shocks applied in the section.
2. Subtract 1 from each of the values obtained in step 1. This gives the number of boat movements between shocks.
3. Multiply the values from step 2 with the boat length and multiply by either 1 or 2 if either one or two boat length are moved between each shock). This gives the unfished distance of each section.
4. Subtract the values obtained in step 3 from the total length of the sections to obtain the effective fished distance per section.
5. Summarise values from all sections and multiply with the effective fished width to obtain the effective fished area.

## Caveats

**Fig. 2:**
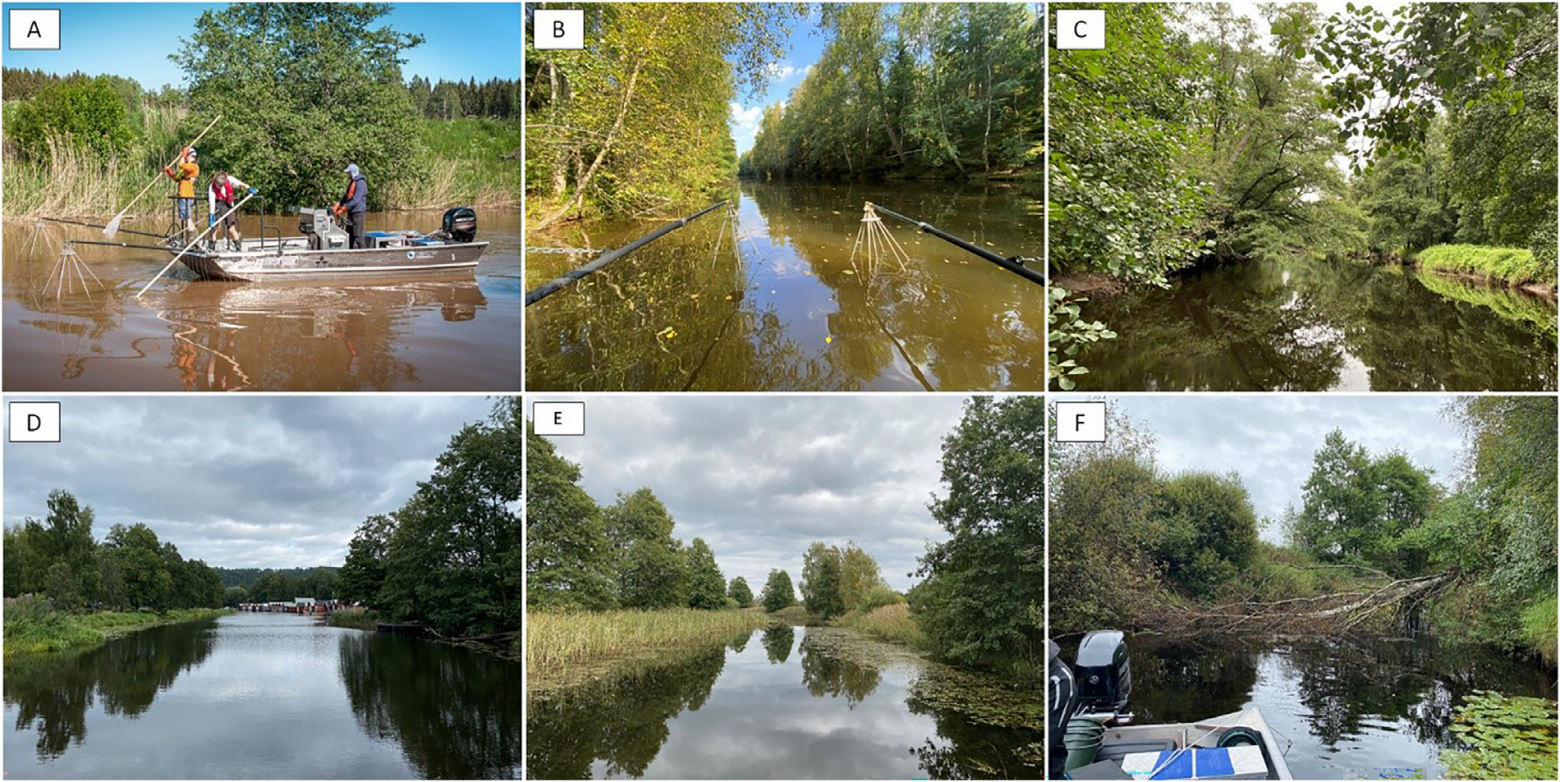
Examples of Swedish slow-flowing river types where the method is applicable, showing a variety of water colour, aquatic vegetation and riparian environments. A) Sagån (Norrström catchment). B) Stångån (Motala ström catchment). C) Suseån (Suseån catchment). D) Huskvarnaån (Motala ström catchment). E) Solgenån (Emån catchment). F) Kättarpsån (Ätran catchment); river channel blocked by fallen tree. Photo credits: A) J. Näslund; B-F) T. Staveley.

The presented method is assessed to work well in medium-sized (up to ca. 50 m width) slow-flowing rivers in southern and central Sweden [2] (Fig. 2). In a few cases, this method been tested in some larger rivers, though the catches have been very low [2]. One way to mitigate this problem of low catches might be to conduct fishing over longer stretches (i.e. longer sites being several kilometres). Although, this remains to be tested systematically.

Fishing in faster-flowing waters (current velocities > 0.3 m · s^−1^) has not yet been evaluated (but data collection started in 2023). Fishing in faster flows in shallow areas typically means that the intermittent operation can be hard to realise; initial tests indicate that continuous power output could work well and be more suitable in these types of river sections. The suitability of the standardised method for this type of fishing needs further systematic testing and the method will likely be amended in the future.

Due to the focus on shoreline sampling, the method is not developed for species inventory surveys (see [9]), quantitative stock-/population assessment, or to target specific species. For these purposes, other operation strategies should be applied. For more complete species inventories, it is recommended that alternative sampling methods (e.g. eDNA sampling, gill nets, or fyke nets) are supplementing the electrofishing.

For status assessment, no assessment index is yet developed for Swedish conditions (as of August 2024). Assessment based on boat electrofishing therefore needs to rely on expert judgement.

The method is likely applicable in other countries, but might require adjustments of recommendations for site- and section lengths and shock durations, depending on specific environmental conditions, fish abundance, and fish community composition. Hence, we recommend systematic testing of the method before applying it in a wide scale outside of Sweden. Furthermore, the collected metadata may need to be adjusted or amended, since environments could differ significantly from the typical Swedish conditions represented in Table 1. Finally, it should be ascertained that the method follows national legislation and fisheries regulations; if not it should obviously be adapted accordingly.

## Concluding remarks

This paper describes the first version of a Swedish standardised boat electrofishing method. While being officially published in Swedish in 2022 [1], the method has been in operation since 2021 for the purpose of developing national monitoring assessment indices and for research projects [e.g. comparisons between boat electrofishing and eDNA metabarcoding (Näslund et al. *in prep*.)]. As a consequence of the fact that the method still remains untested in some types of rivers, the current method should primarily be seen as a method for medium-sized slow-flowing rivers. With further assessment under different conditions, the method may be amended (i.e. readers of this article are advised to search for updates to the method).

### Ethics statements

For test fishing surveys during method development, fish were electrofished under an ethical permit for animal research issued by the Swedish Board of Agriculture (Dnr 6229–2020; reviewed by the Stockholm ethical committee). All fishing rights owners provided permits to fish in their respective river sections. Electrofishing permits were issued by the Department of Aquatic Resources, Swedish University of Agricultural Sciences, based on an exemption from national fisheries regulations for the purpose of environmental monitoring, granted to the department from the Swedish Agency for Marine and Water Management. All involved personnel had the required education in animal ethics and electrofishing.

## CRediT authorship contribution statement

**Joacim Näslund:** Project administration, Conceptualization, Methodology, Data curation, Investigation, Validation, Supervision, Visualization, Writing – original draft, Writing – review & editing. **Thomas A.B. Staveley:** Project administration, Investigation, Data curation, Supervision, Writing – review & editing. **Erik Petersson:** Project administration, Conceptualization, Methodology, Investigation, Writing – review & editing.

## Declaration of comepting interest

The authors declare that they have no known competing financial interests or personal relationships that could have appeared to influence the work reported in this paper.
